# Activity of the response regulator CiaR in mutants of *Streptococcus pneumoniae* R6 altered in acetyl phosphate production

**DOI:** 10.3389/fmicb.2014.00772

**Published:** 2015-01-15

**Authors:** Patrick Marx, Marina Meiers, Reinhold Brückner

**Affiliations:** Department of Microbiology, University of KaiserslauternKaiserslautern, Germany

**Keywords:** *Streptococcus pneumoniae*, two-component regulatory system CiaRH, acetyl phosphate, alternative phosphorylation, pyruvate oxidase, acetate kinase

## Abstract

The two-component regulatory system (TCS) CiaRH of *Streptococcus pneumoniae* is implicated in competence, ß-lactam resistance, maintenance of cell integrity, bacteriocin production, host colonization, and virulence. Depending on the growth conditions, CiaR can be highly active in the absence of its cognate kinase CiaH, although phosphorylation of CiaR is required for DNA binding and gene regulation. To test the possibility that acetyl phosphate (AcP) could be the alternative phosphodonor, genes involved in pyruvate metabolism were disrupted to alter cellular levels of acetyl phosphate. Inactivating the genes of pyruvate oxidase SpxB, phosphotransacetylase Pta, and acetate kinase AckA, resulted in very low AcP levels and in strongly reduced CiaR-mediated gene expression in CiaH-deficient strains. Therefore, alternative phosphorylation of CiaR appears to proceed via AcP. The AcP effect on CiaR is not detected in strains with CiaH. Attempts to obtain elevated AcP by preventing its degradation by acetate kinase AckA, were not successful in CiaH-deficient strains with a functional SpxB, the most important enzyme for AcP production in *S*. *pneumoniae*. The *ciaH*-*spxB*-*ackA* mutant producing intermediate amounts of AcP could be constructed and showed a promoter activation, which was much higher than expected. Since activation was dependent on AcP, it can apparently be used more efficiently for CiaR phosphorylation in the absence of AckA. Therefore, high AcP levels in the absence of CiaH and AckA may cause extreme overexpression of the CiaR regulon leading to synthetic lethality. AckA is also involved in a regulatory response, which is mediated by CiaH. Addition of acetate to the growth medium switch CiaH from kinase to phosphatase. This switch is lost in the absence of AckA indicating metabolism of acetate is required, which starts with the production of AcP by AckA. Therefore, AckA plays a special regulatory role in the control of the CiaRH TCS.

## Introduction

The two-component regulatory system (TCS) Competence Induction and Altered cefotaxime susceptibility (CiaRH) of the human pathogen *Streptococcus pneumoniae* is a pleiotropic regulatory device, which is involved in controlling a variety of physiological processes such as genetic competence (Sebert et al., 2005; Cassone et al., 2012), maintenance of cell integrity (Dagkessamanskaia et al., 2004; Mascher et al., 2006a), ß-lactam resistance (Guenzi et al., 1994; Müller et al., 2011), bacteriocin production (Dawid et al., 2009; Kochan and Dawid, 2013), and virulence (Throup et al., 2000; Sebert et al., 2002; Ibrahim et al., 2004). CiaR belonging to the OmpR family of response regulators (Martínez-Hackert and Stock, 1997) and the periplasmic-sensing EnvZ-PhoQ-type (Mascher et al., 2006b) histidine kinase CiaH constitute a prototypical TCS. The response regulator CiaR controls directly 16 promoters, 15 positively and one negatively (Halfmann et al., 2007b; Denapaite et al., 2012). These promoters drive transcription of 29 genes, among them five genes specifying small non-coding RNAs (csRNAs, cia-controlled small RNAs) (Halfmann et al., 2007b). The csRNAs in turn are implicated in autolysis (Halfmann et al., 2007b), competence (Tsui et al., 2010; Schnorpfeil et al., 2013), ß-lactam resistance (Schnorpfeil et al., 2013), and virulence (Mann et al., 2012). In addition, a gene product of another member of the CiaR regulon, the serine protease HtrA, participates in regulating most of the above mentioned phenotypes (Ibrahim et al., 2004; Cassone et al., 2012; Kochan and Dawid, 2013).

TCSs with transcription regulators as output domains typically turn gene expression on and off in response to the presence of certain stimuli (Gao and Stock, 2009; Krell et al., 2010). CiaRH however, was found to be highly active under a variety of laboratory growth conditions (Halfmann et al., 2011), but also in animal models analyzing colonization and virulence (Throup et al., 2000; Marra et al., 2002; Sebert et al., 2002; Ibrahim et al., 2004). Moreover, the system is active in various *S*. *pneumoniae* clinical isolates (Sebert et al., 2002; Lanie et al., 2007; Kumar et al., 2010; Tsui et al., 2010), not only in strain R6 used in this study.

Even in the absence of CiaH, CiaR-dependent promoters are still active and their strength may even be higher than with a functional CiaH under certain growth conditions (Halfmann et al., 2011). On the other hand, purified CiaR did not bind to promoter fragments unless acetyl phosphate (AcP) was added prior to the gel shift assay (Halfmann et al., 2011). A mutant form of CiaR containing an alanine instead of aspartic acid at position 51 was no longer able to activate gene expression and did not bind efficiently to promoter fragments even in the presence of AcP (Halfmann et al., 2011). Therefore, *in vitro* and *in vivo* evidence strongly suggests that the phosphorylated form of CiaR is active in DNA binding and transcriptional regulation. Nevertheless, high CiaR-dependent promoter activity is detected in the absence of CiaH indicating that CiaR must be able to obtain its phosphate from other sources.

Two ways are conceivable how CiaR could be phosphorylated in the absence of cognate CiaH kinase: Phosphorylation by another histidine kinase by cross-talk (Laub and Goulian, 2007), or cross-phosphorylation by small molecular weight high-energy donors such as AcP (Lukat et al., 1992; Wanner, 1992; Wolfe, 2010). Since *in vitro* CiaR DNA binding activity could be stimulated by AcP, CiaR may indeed be phosphorylated by this molecule *in vivo*.

Synthesis of AcP in *S*. *pneumoniae* depends on three enzymes, pyruvate oxidase SpxB, phosphotransacetylase Pta, and acetate kinase AckA (Spellerberg et al., 1996; Pericone et al., 2003; Ramos-Montanez et al., 2010; Carvalho et al., 2013). SpxB uses oxygen and inorganic phosphate to produce AcP and H_2_O_2_ directly from pyruvate. AcP production by Pta proceeds via acetyl-coenzyme A, which is produced by pyruvate-formate lyase (Yesilkaya et al., 2009). Finally, AckA converts AcP to acetate, which is excreted, and generates ATP. The enzyme is also able to catalyze the reverse reaction but the equilibrium is far toward ATP formation.

In the present study, we examined the hypothesis that AcP could be the phosphoryl donor for CiaR by creating mutants in the *spxB, pta*, and *ackA* AcP biosynthesis genes and subsequent measurements of CiaR-mediated gene expression in the presence or absence of CiaH. The results of these experiments provide strong evidence that AcP is important for CiaR phosphorylation in the absence of CiaH. They also reveal a special role of AckA in this regulation.

## Materials and methods

### Bacterial strains, plasmids, growth conditions, and transformation

The *S. pneumoniae* strains used in this study are derivatives of *S. pneumoniae* R6 (Ottolenghi and Hotchkiss, 1962) and are listed in Table 1. *S. pneumoniae* was grown at 37°C in static cultures without aeration in C-medium (Lacks and Hotchkiss, 1960) supplemented with 0.1% yeast extract (C+Y) or brain-heart infusion (BHI). BHI was purchased from Becton Dickinson, France. The cultures for all experiments had a volume of 10 ml and were kept in glass tubes with a diameter of 1.4 mm. Therefore, *S. pneumoniae* was grown under semi-aerobic conditions, identical to our previous experiments. For the acetate experiments, C+Y-medium and BHI-medium have been modified. The standard C+Y-medium contains 12.5 mM sodium acetate (Lacks and Hotchkiss, 1960). Modified C+Y medium contained no sodium acetate, 25 mM or 50 mM. Sodium acetate was added from a 3M stock solution, which had been adjusted to pH 7.8, the pH of C+Y medium. BHI was treated similarly, except that a 3M sodium acetate stock solution of pH 7.4 was used according to the pH of this medium. Growth of *S. pneumoniae* was monitored by measuring optical density of 600 nm (OD_600_). *S. pneumoniae* and its derivatives were grown at 37°C on plates containing D-blood agar (Alloing et al., 1996). Strains with inactivated *ackA* were kept in an air proof candle jar containing 5% oxygen and 10% carbon dioxide.

**Table 1:** ***S. pneumoniae* strains used in this study**.

**Strain^a^**	**Characteristics**	**Sources or references**
R6	Wild type	Ottolenghi and Hotchkiss, 1962
RCH	*ciaH*::*aad9*	Halfmann et al., 2011
RKL95	*spxB*::*ermB*	This work
RKL399	*spxB*::*ermB, pta*::*cat*	This work
RKL369	*spxB*::*ermB, pta*::*cat, ackA*::*aphIII*	This work
RKL394	*spxB*::*ermB, ackA*::*aphIII*	This work
RKL380	*pta*::*cat*	This work
RKL416	*pta*::*cat, ackA*::*aphIII*	This work
RKL379	*ackA*::*aphIII*	This work
RKL400	*ciaH*::*aad9, spxB*::*ermB*	This work
RKL410	*ciaH*::*aad9, spxB*::*ermB, pta*::*cat*	This work
RKL373	*ciaH*::*aad9, spxB*::*ermB, pta*::*cat, ackA*::*aphIII*	This work
RKL401	*ciaH*::*aad9, spxB*::*ermB, ackA*::*aphIII*	This work
RKL385	*ciaH*::*aad9, pta*::*cat*	This work
RKL168	*ciaH306, rpsL41*^b^	Müller et al., 2011
RKL162	*ciaH202, rpsL41*	Müller et al., 2011
RKL163	*ciaH305, rpsL41*	Müller et al., 2011
RKL164	*ciaH208, rpsL41*	Müller et al., 2011
RKL165	*ciaH408, rpsL41*	Müller et al., 2011
RKL243	*ciaH232, rpsL41*	Müller et al., 2011
RKL245	*ciaH556, rpsL41*	Müller et al., 2011
RKL246	*ciaH1057, rpsL41*	Müller et al., 2011
RKL244	*ciaH*_TpVT_*, rpsL41*	Müller et al., 2011

a *All strains are R6 derivatives*.

b *Strains contain rpsL41 (strepR) because ciaH alleles were introduced by the Janus counter-selection procedure (Sung et al., 2001)*.

The promoter probe plasmids with *E. coli lacZ* as a reporter gene pPP2 containing *htrA*, spr0931, and *ccnA* promoters, respectively, were described previously (Halfmann et al., 2007b). The plasmids were multiplied in *E. coli* DH5α [ϕ80d*lacZ*ΔM15Δ(*lacZYA*-*argF*) *recA1 endA1 hsdR17 supE44 thi-1 gyrA96 phoA relA1*] and subsequently transferred to *S*. *pneumoniae* strains as described (Halfmann et al., 2007b). Natural competent cells of *S. pneumoniae* were only used for the wild type R6 and the *ciaH* mutant RCH. The other mutant strains described in this study were transformed by the addition of CSP as described (Schnorpfeil et al., 2013), since their competence development has not been thoroughly studied. *E. coli* strains were grown in LB medium and transformed according to Hanahan (1983).

### Gene inactivations

The oligonucleotides used for the gene inactivations are listed in Table 2. The construction of the *ciaH* mutant (*ciaH*::*aad9*) has been described previously (Halfmann et al., 2011). The *spxB* inactivation construct was obtained in strain *S. pneumoniae* D39 and its construction has been described elsewhere (Bättig and Mühlemann, 2008). The *spxB* gene inactivated by an erythromycin resistance gene (*spxB*::*ermB*) was amplified by primer pair spxB_fwd2, spxB_rev2 and transferred to *S. pneumoniae* R6 and its derivatives by selection on 0.5 μg/μl erythromycin. Integration was confirmed by PCR. The *spxB* mutant of R6 was designated RKL95 (Table 1).

**Table 2:** **Oligonucleotides used in this study**.

**Primer^a^**	**Sequence**
spxB_fwd2	CGGTTCAGGTTCATACGAACGCTC
spxB_rev2	CAACTGGGTTTACTTTGTCAAGG
cat1	GCGACTAGTTTGGACTCCTGTTGATAGATCC
cat2	GGCGCATGCACAAAAAATGGACTGAACAAGTCAG
ptaf1	CGCAGATGAACATGTCAAGG
ptar1	GCGACTAGTCTTCCATGAGTTTTCTCCTTTAAG
ptaf2	GCGGCATGCGGAGCGATTCACTCAACAGC
ptar2	GGGAATTTATCGTTTTACGGAC
ptaf3	GGAGACGGGCTAACAGTTTC
ackAf2B	GCGGGATCCGAAAAGAGGAAGGAAATAATAAATGG
ackAr2B	GCGGCATGCATTCTCAGGCACCAAGCCC
ackAf1	TCTGGATGGTGAAACTGAGC
ackAr1B	GCGGGATCCTAACTGATACCCCTTTTAAGC
ackAf3B	GCGGCATGCAGTGAAACTAAAAAAATATTCAATAC
ackAr3	CCTGGATATTGTTCTCGAAGC
ackAf4	GTGGAGCAGCAAGCAATCAAG
ackAr4	TGTGCCTGTACTACGGCCTCG

a *Recognition sites for restriction enzymes are underlined*.

To construct the *pta* mutant, a direct PCR cloning approach has been performed to replace nucleotides 9 to 366 of the *pta* coding region by the chloramphenicol resistance cassette *cat*. The *pta* coding region consists of 974 bp and is followed immediately downstream by two IS-elements (Hoskins et al., 2001). To allow specific homologous recombination, the 3′-part of *pta* was kept. The *cat* gene fragment was generated by primer pair cat1, cat2 using plasmid puc18-VegM-CAT2 as template (Halfmann et al., 2007b). The upstream fragment of *pta* was amplified by primers ptaf1, ptar1, the downstream fragment containing the 3′-end of *pta* by primers ptaf2, ptar2. Each of the primers incorporate recognition sequences for the restriction enzymes *Sph*I and *Spe*I. The amplicons were ligated subsequent to their cleavage. The ligation was re-amplified by primer pair ptaf1 and ptar2 and *S*. *pneumoniae* R6 transformants were selected on 3 μg/ml chloramphenicol to obtain strain RKL380 (*pta*::*cat*). Integration was confirmed by PCR and sequencing. Subsequently, DNA of strain RKL380 was used as template to amplify the *pta*::*cat* deletion construct with primers ptaf3 and ptar2. The amplicon was then used to move the *pta* deletion to different *S*. *pneumoniae* strains.

The *ackA* mutant has been constructed by replacement of the entire gene coding region with the kanamycin resistance gene *aphIII* (Halfmann et al., 2007b). The *aphIII* fragment was generated with primers ackAf2B and ackAr2B. The upstream fragment of *ackA* was amplified with ackAf1 and ackAr1B while the downstream fragment was amplified with primers ackAf3B and ackAr3. Each of the primers contained recognition sequences for the restriction enzymes *SphI* and *BamHI*. The amplicons were ligated subsequently to their cleavage. The entire ligation was amplified using primer pair ackAf1, ackA3r. Since problems have been reported inactivating *ackA* in *S*. *pneumoniae* D39 (Ramos-Montanez et al., 2010), this fragment was transferred first to the *spxB* mutant strain RKL95. Selection occurred on 100 μg/ml kanamycin containing blood agar plates incubated in an atmosphere with reduced oxygen (5%) and 10% CO_2_. Integration was confirmed by PCR and sequencing. Chromosomal DNA of this strain was then used to amplify the *ackA*::*aphIII* fragment to transform *S. pneumoniae* R6 to yield strain RKL379 and the other *ackA* deletion strains (Table 1).

Inactivation of *ackA* was generally performed as the latest step in strain constructions. In the presence of *ciaRH*, no problems were encountered during *ackA* inactivation. Since genetic instability of *ackA* mutants was described for D39, the predecessor of R6, the genes found to be mutated in this study, *spxB* and *spxR* (Ramos-Montanez et al., 2010), were sequenced in several colonies of our *ackA* mutant strains. No alterations were detected. In the *ciaH*::*aad9* mutant strain however, *ackA* inactivation could not be achieved in the presence of a functional SpxB. The transformation efficiency with the *ackA*::*aphIII* fragment dropped in RCH (*ciaH::aad9*) or in RKL385 (*ciaH*::*aad9, pta*::*cat*, Table 1) by about four orders of magnitude compared to the corresponding strains with *ciaRH* (R6, RKL80, Table 1) or strains with inactivated *spxB* (RKL400, RKL410, Table 1). Since the strains with intact *spxB* that could not be obtained efficiently produced large amounts of H_2_O_2_, katalase was added to the agar. However, transformation efficiency did not improve. The bacteria recovered in these transformations were subsequently found to have suppressor mutations in *spxR*, but also in *ciaR*.

Mutations in *ciaR* were intriguing, since it suggested that an intact *ciaR* is detrimental in this genetic constellation. And indeed, a *ciaR* mutant could be efficiently transformed with the *ackA*::*aphIII* fragment. Sequencing *spxB* in a couple of transformants revealed no mutations. Thus, inactivation of *ciaH* and *ackA* causes synthetic lethality, which can be suppressed by mutation of *spxB*, but also by *ciaR* inactivation.

Since the non-coding csRNAs and the protease HtrA were implicated in regulating various processes in *S. pneumoniae* (Sebert et al., 2002; Dawid et al., 2009; Schnorpfeil et al., 2013), inactivation of these genes were tested for suppression of the lethal phenotype. Deletion of the csRNA genes had no effect, but *htrA* inactivation resulted in about 200-fold more transformants. Deletion of the csRNA genes and *htrA* together further improved transformation tenfold. However, these transformants also acquired suppressor mutations in *spxB* and *ciaR*.

### Determination of AcP amounts

Determination of cellular levels of AcP was carried out according to described methods (Prüss and Wolfe, 1994; Pericone et al., 2003). All steps have been performed on ice or at 4°C, respectively. Ten ml cultures of the desired strains have been grown to mid-exponential phase (OD_600_ 0.4) and subsequently centrifuged at 9000 rpm for 10 min at 4°C. Cell pellets were resuspended in 1 ml ice cold luciferase buffer (100 mM Tris, 4 mM EDTA, pH 7.6) and transferred into 1.5 ml tubes. The cells were lysed for 2 min at 95°C. Within that time, no degradation of AcP was observed in tests using purified AcP (Sigma Aldrich). The cells were quickly cooled on ice, kept for 10 min and then centrifuged at 10,000 rpm for 2 min at 4°C. The supernatant was transferred to a 2 ml tube and mixed with 50 mg/ml powdered activated charcoal (Sigma Aldrich) to remove small compounds like ADP and ATP. After incubation on ice for 15 min, the samples have been filtered (0.22 μm pore-size) to remove the charcoal. The remaining AcP in the samples was enzymatically converted into ATP by acetate kinase. Samples of 100 μl were mixed with the following compounds in the indicated final concentration: 20 mM MgCl_2_, 30 mM ADP (Sigma Aldrich), 0.5 U/μl acetate kinase from *B. stearothermophilus* (Sigma Aldrich). Samples containing acetate kinase have been prepared as duplicates and one sample was prepared without acetate kinase as negative control and a control for ATP removal as well. The reaction mixtures were incubated at 30°C for 4 h. The generated ATP was determined by measuring luminescence using the ATP Bioluminescence Assay Kit CLS II (Roche) following the manufacturers' instructions. Luciferase Reagent was added to the acetate kinase reaction mixtures at the ratio of 1. Samples were incubated at room temperature for 2 min and ATP was determined in a BioOrbit 1253 luminometer. Data obtained from samples with acetate kinase were took as means and subtracted from data obtained from samples without acetate kinase. ATP amounts were determined by comparison with standard curves. Standard curves were generated using known AcP amounts in concentrations between 0.2 and 50 μM, which were converted to ATP as described above. Protein content of the samples was determined as described (Halfmann et al., 2007a) to relate AcP levels to protein.

### Determination of ß-galactosidase activity

To assess the activity of CiaR, strains harboring CiaR-dependent promoter-ß-galactosidase gene (*lacZ*) fusions in the genome (Halfmann et al., 2007b) were assayed for ß-galactosidase activity as described (Halfmann et al., 2007a). Units are expressed in nmol nitrophenol released per min and mg of protein.

## Results

### Acetyl phosphate levels in *s. pneumoniae* R6 and mutants with altered pyruvate metabolism

**Figure 1:**
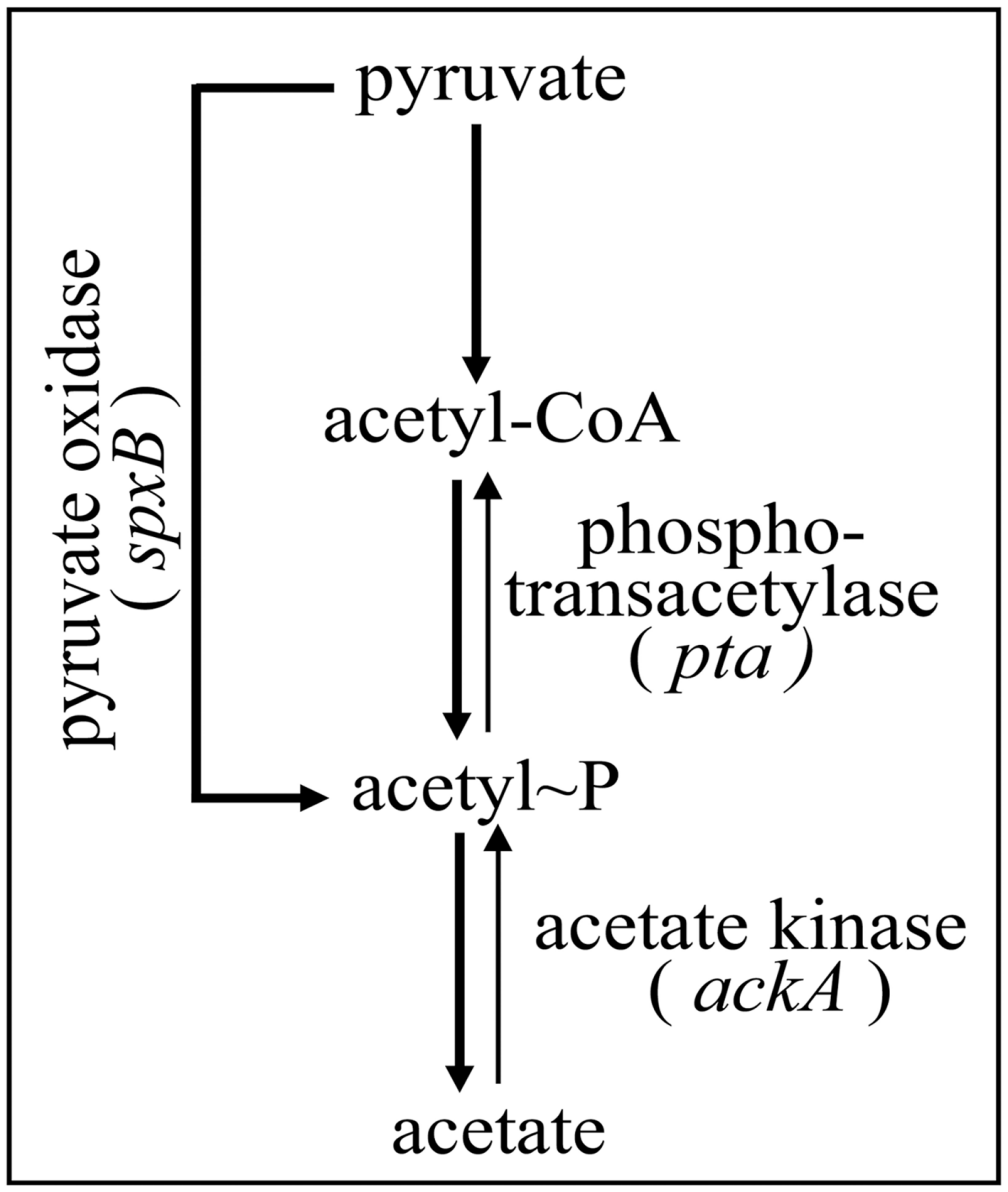
**Acetyl phosphate production in *S. pneumoniae***. The part of pyruvate metabolism relevant for this study is shown. Three enzymes, SpxB, Pta, and AckA, are implicated in AcP production as detailed in the introduction. Acetyl-CoA is produced from pyruvate by pyruvate formate lyase (Yesilkaya et al., 2009).

**Figure 2:**
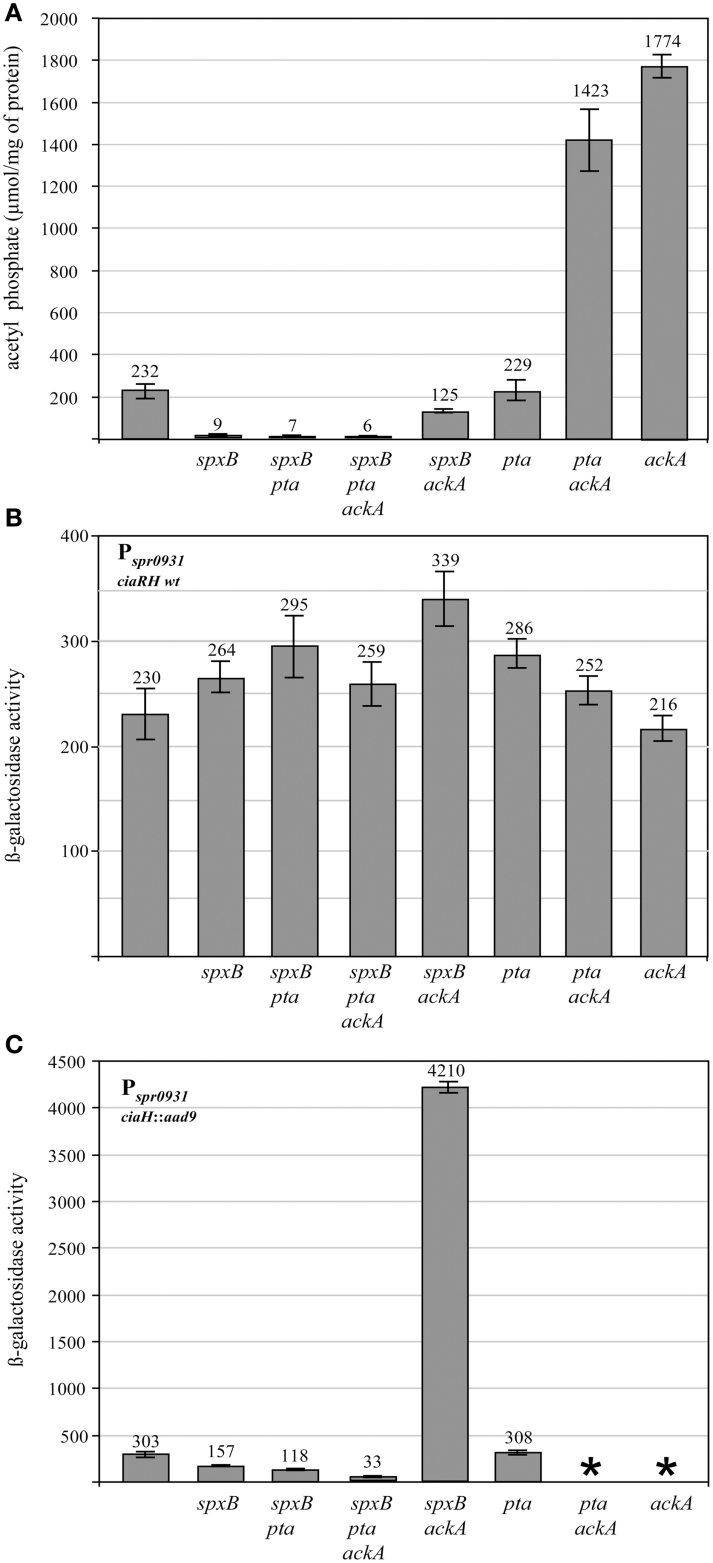
**Acetyl phosphate levels and promoter activities in pyruvate metabolism mutants of *S. pneumoniae* R6. (A)** Acetyl phosphate in pyruvate metabolism mutants. The strains were grown in C+Y medium to mid-expontential growth phase (OD_600_ 0.4) and acetyl phosphate was determined from 10 ml of the cultures. The strains used in these determinations were (from left to right): R6, RKL95 (*spxB*::*ermB*), RKL399 (*spxB*::*ermB, pta*::*cat*), RKL369 (*spxB*::*ermB, pta*::*cat, ackA*::*aphIII*), RKL394 (*spxB*::*ermB, ackA*::*aphIII*), RKL380 (*pta*::*cat*), RKL416 (*pta*::*cat, ackA*::*aphIII*), RKL379 (*ackA*::*aphIII*). All strains are derivatives of R6 and harbor wild type *ciaRH*. The inactivated genes are indicated. AcP was determined in at least two independent cultures and the values are shown along with standard deviations. **(B)** CiaR-dependent promoter activity in pyruvate metabolism mutants harboring *ciaRH* wild type. The strains were grown in C+Y medium to an OD_600_ of 0.4 and ß-galactosidase activity was determined. The strains were (from left to right): R6, RKL95 (*spxB*::*ermB*), RKL399 (*spxB*::*ermB, pta*::*cat*), RKL369 (*spxB*::*ermB, pta*::*cat, ackA*::*aphIII*), RKL394 (*spxB*::*ermB, ackA*::*aphIII*), RKL380 (*pta*::*cat*), RKL416 (*pta*::*cat, ackA*::*aphIII*), RKL379 (*ackA*::*aphIII*). All strains contained a P_spr0931_ promoter *lacZ* fusion in the genome (*bgaA*::*tetM*-P_spr0931_-*lacZ*). The promoter of spr0931 is activated by CiaR and its activity is strictly CiaR-dependent. Two other promoters, P*_htrA_* and P*_ccnA_*, reacted as P_spr0931_and a CiaR-independent promoter P*_vegM_* showed no change in activity. The inactivated genes are indicated. Values of at least three independent cultures are shown along with standard deviations. ß-galactosidase units are expressed in nmol nitrophenol released per min and mg of protein. (**C)** CiaR-dependent promoter activity in pyruvate metabolism mutants harboring inactivated *ciaH*. The strains were (from left to right): RCH (*ciaH*::*aad9*), RKL400 (*ciaH*::*aad9, spxB*::*ermB*), RKL410 (*ciaH*::*aad9, spxB*::*ermB, pta*::*cat*), RKL373 (*ciaH*::*aad9, spxB*::*ermB, pta*::*cat, ackA*::*aphIII*), RKL401 (*ciaH*::*aad9, spxB*::*ermB, ackA*::*aphIII*), RKL385 (*ciaH*::*aad9, pta*::*cat*). ^*^Indicates that these strains could not be constructed. All strains had a P_spr0931_ promoter *lacZ* fusion in the genome (*bgaA*::*tetM*-P_spr0931_-*lacZ*). The promoter of spr0931 is shown as a representative for CiaR-dependent promoters. The control promoter P*_vegM_* was not affected. All strains are CiaH-deficient. The inactivated genes are indicated. Values of at least three independent cultures are shown along with standard deviations. ß-galactosidase units are expressed in nmol nitrophenol released per min and mg of protein.

Since the enzymes pyruvate oxidase SpxB, phosphotransacetylase Pta, and acetate kinase AckA are the key determinants for AcP production in *S. pneumoniae* (Figure 1), inactivation of their genes should substantially alter AcP levels. Therefore, the respective genes were inactivated by resistance cassettes alone or in combination as described in Materials and Methods. To start with this genetic analysis the wild type strain R6 was used carrying an intact *ciaRH* system. The mutants were grown in C+Y medium, the medium supporting high levels of CiaR activity in the absence CiaH (Halfmann et al., 2011), and AcP was measured as described in Materials and Methods. As shown in Figure 2A, pyruvate oxidase SpxB is the major enzyme involved in AcP production, a result consistent with earlier work (Pericone et al., 2003; Ramos-Montanez et al., 2008, 2010). The AcP level in the *spxB* mutant of R6 strain dropped about 20-fold, which is a larger reduction than in *S. pneumoniae* D39 (Pericone et al., 2003; Ramos-Montanez et al., 2008, 2010). This difference is most likely the consequence of an *spxB* polymorphism detected in both strains (Belanger et al., 2004; Ramos-Montanez et al., 2008). The enzyme is apparently more active in R6 (Belanger et al., 2004). Single inactivation of *pta* encoding phosphotransacetylase had no significant effect, but without SpxB and acetate kinase AckA a contribution of Pta to AcP production is detectable. Inactivation of *ackA* in an SpxB-deficient strain increased the low level of AcP about 14-fold (Figure 2A), because conversion of AcP to acetate by AckA is blocked and AcP can accumulate (Wolfe, 2005). In the *spxB-pta* double mutant however, *ackA* mutation did not result in the elevation of AcP. Thus, some AcP had been produced by Pta in the absence of SpxB. Why Pta is not significantly contributing to AcP production in the presence of SpxB is not clear at the moment.

Growth was differently affected in the mutant strains (Table S1). The single mutants in *spxB* or *ackA* grew almost like the wild type, all other mutant strains were slower with doubling times ranging between 45 and 55 min compared to 37 min of the wild type.

The AcP determinations in this series of *S. pneumoniae* R6 pyruvate metabolism mutants largely confirmed what could be expected from the literature (Pericone et al., 2003; Wolfe, 2005; Ramos-Montanez et al., 2010). Without SpxB and Pta, AcP production is virtually absent. AcP produced by SpxB or Pta accumulates, when *ackA* is inactivated. Consequently, R6 derivatives are available covering a wide range of AcP levels. Since the AcP synthesis mutants were constructed to analyze the connection of AcP to CiaRH-mediated gene expression, it was of interest to determine whether CiaRH could influence AcP levels. AcP determinations in CiaR-, and CiaH-deficient as well as in CiaRH hyperactive strains (Müller et al., 2011) did not reveal an influence (Figure S1).

### CiaR-mediated gene expression in AcP synthesis mutants in the presence of CiaH

During the initial characterization of the CiaR regulon (Halfmann et al., 2007b), all CiaR-controlled promoters were cloned into an integrative reporter plasmid (Halfmann et al., 2007a). These constructs are well-suited to analyze CiaR-dependent gene expression (Halfmann et al., 2007b; Müller et al., 2011). Therefore, three of these promoter fusions, P_spr0931_, P*_htrA_*, and P*_ccnA_*, were introduced into the mutant strains described above as representatives of the CiaR regulon. They were chosen because their activity is strongly dependent on CiaR and there is no evidence that they are controlled by other regulators (Halfmann et al., 2007b). Measuring the promoter activities in C+Y medium revealed only subtle changes in an almost identical range for all promoters (data not shown). As a representative example, P_spr0931_ mediated ß-galactosidase expression determined in the middle of exponential growth (OD_600_ 0.4) is shown in Figure 2B. In general, promoter activities are slightly higher in almost all mutant strains. The *ackA*-*pta* mutant showed the strongest activation with an increase of 1.5-fold. Similar results were obtained measuring earlier (OD_600_ 0.2) or later (OD_600_ 0.8). Considering that AcP levels vary more than 100-fold (Figure 2A), one can conclude that CiaR-dependent promoters are not influenced by AcP under these conditions. The strains used in this series of experiments carried wild type *ciaRH*. Therefore, CiaH will predominantly control the phosphorylation status of CiaR and thereby promoter activities.

### CiaR-mediated gene expression in AcP synthesis mutants in the absence of CiaH

Since cross-phosphorylation or cross-talk in TCSs is quite often only detected in the absence of cognate histidine kinases (Laub and Goulian, 2007), it was of interest to determine promoter activities in the AcP synthesis mutants without CiaH. And indeed, drastic changes were detected in the *ciaH* mutant strains (Figure 2C), quite in contrast to the strains carrying *ciaRH* wild type (Figure 2B). Inactivation of *spxB* resulted in a twofold reduction of P_spr0931_ activity. The other CiaR-dependent promoters reacted accordingly (data not shown). While *pta* inactivation in the *spxB* mutant resulted in a further 2.5-fold reduction, promoter activity dropped about tenfold in the *spxB*-*pta*-*ackA* triple mutant. Therefore, inactivation of the genes of pyruvate metabolism, which resulted in the reduction of AcP (Figure 2A) clearly diminished CiaR-dependent transcription. Accordingly, no change was observed in the *pta* mutant (Figure 2C), which had wild type AcP levels (Figure 2A).

A surprising result, however, has been obtained in the *spxB*-*ackA* mutant strain (Figure 2C). Although AcP is about twofold reduced compared to the wild type (Figure 2A), transcription from CiaR-dependent promoters is tremendously increased. P_spr0931_ activity rose about 14-fold (Figure 2C). In addition, inactivation of *ackA* in strains with intact *spxB*, which are characterized by a high amount of AcP (Figure 2A) could not be achieved. Disruption of the same genes was possible in strains with a functional CiaH, but also in the absence of CiaR (see Materials and Methods). Considering the very strong P_spr0931_activation in the *spxB*-*ackA*-*ciaH* mutant strain, it appears that the inability to introduce mutations in *ackA* to strains with a functional SpxB in the absence of CiaH is caused by extreme activation of the CiaR regulon. We have shown previously that strong activation of the CiaR regulon by mutations in *ciaH* could impair growth (Müller et al., 2011). It is therefore conceivable, that higher activation of CiaR-mediated transcription in the AckA-deficient strains could lead to lethality.

The results of these experiments clearly demonstrate a strong influence of the pyruvate metabolism on CiaR-mediated gene expression, provided the cognate kinase CiaH was absent. Promoter activation was strongly reduced in strains producing low amounts of AcP, which would be consistent with AcP as the alternative route of phosphorylation for CiaR in the absence of CiaH. The experiments also show that there is no strict correlation between the AcP level and promoter activation. Especially in the absence of AckA in the *spxB* mutant background, CiaR is disproportionally activated.

### The influence of acetate in the growth medium on CiaR-mediated gene expression

**Figure 3:**
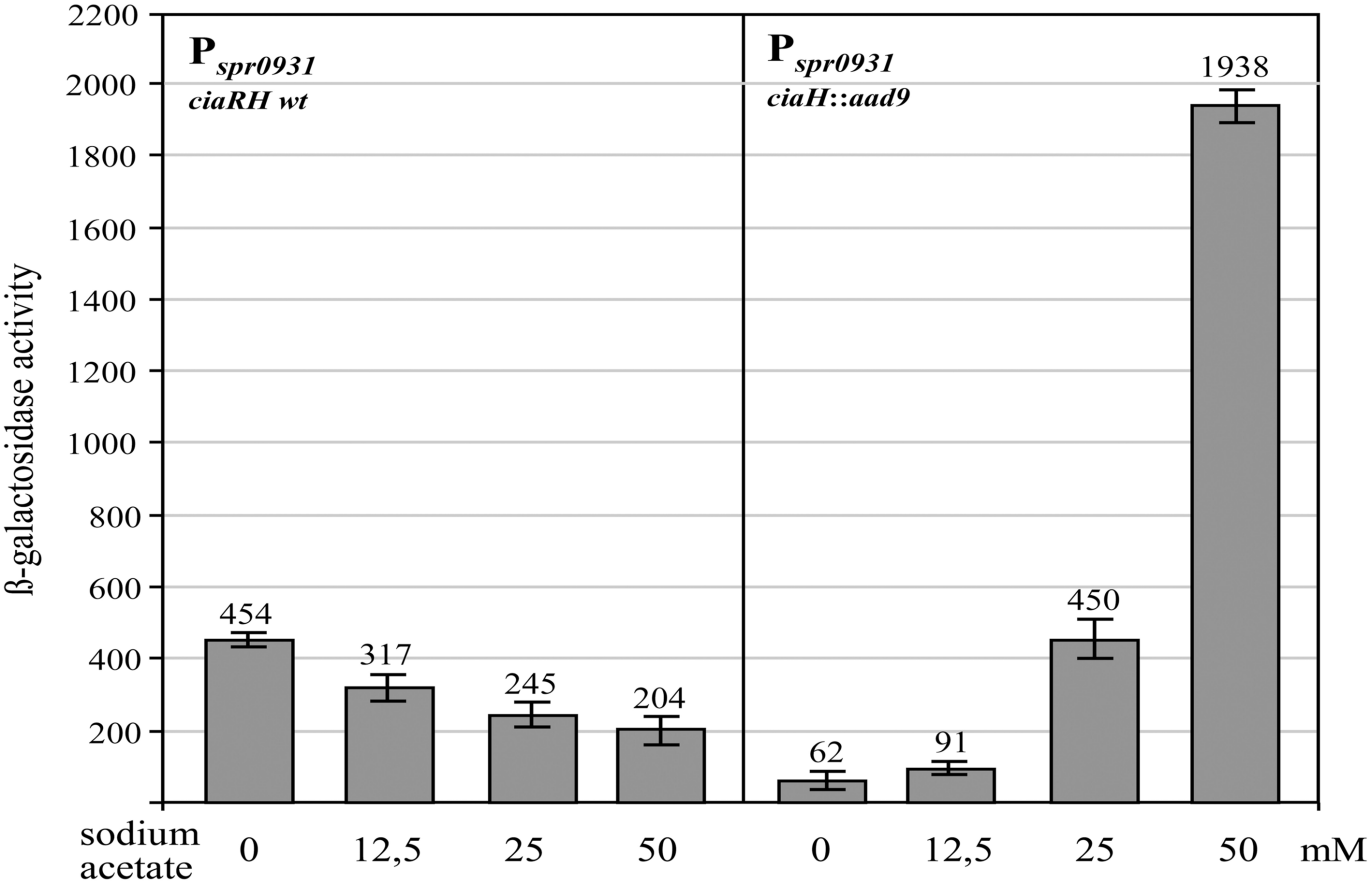
**Effect of acetate supplementation of the growth medium on CiaR-dependent promoter activity**. The strains were grown in BHI medium containing the indicated concentrations of sodium acetate to an OD_600_ of 0.4 and ß-galactosidase was measured. The comparison of the promoter activities of the R6 wild type harboring *ciaRH* (four measurements from the left) with those found in the CiaH-deficient mutant RCH (*ciaH*::*aad9*) is shown. All strains had a P_spr0931_ promoter *lacZ* fusion in the genome (*bgaA*::*tetM*-P_spr0931_-*lacZ*). The concentrations of sodium acetate are indicated. Values of at least three independent cultures are shown along with standard deviations. ß-galactosidase units are expressed in nmol nitrophenol released per min and mg of protein.

In contrast to the growth of *S. pneumoniae* in C+Y medium, which supports high levels of CiaR-dependent transcription in the absence of CiaH, the same promoters are only weakly active in brain heart infusion (BHI) medium (Halfmann et al., 2011). Measuring AcP in BHI medium revealed an almost threefold reduction (81 ± 9 μmol/mg of protein) compared to C+Y medium (Figure 1), again indicating that AcP levels modulate CiaR activity in the absence of CiaH. This medium was therefore well-suited to test if exogenously added acetate could enhance CiaR activation in the absence of CiaH. As shown in Figure 3, P_spr0931_ activity could be nicely stimulated. Adding 12.5 mM sodium acetate, the concentration found in C+Y medium, activation was moderate (1.5 fold), but twofold (25 mM) or fourfold (50 mM) more sodium acetate enhanced the promoter activity seven- and thirtyfold, respectively (Figure 3). The addition of potassium acetate had the same effect, while sodium chloride did not change P_spr0931_ activity (Figure S2). In conclusion, the observed effect appears to be specific for acetate. Repeating these experiments in C+Y medium with altered acetate concentration yielded in principle the same results, but the change in expression was less pronounced (Figure S3). Unexpectedly, measuring AcP in BHI supplemented with acetate, did not reveal a significant increase, not even with the highest concentration.

Another surprising result was obtained measuring promoter activities in the wild type *ciaRH* strain as control. In this strain, addition of acetate to 50mM reduced the promoter activity about twofold compared to the values obtained without acetate supplementation (Figure 3). In comparison with the activity measured without CiaH however, promoter strength is tenfold reduced. This comparison strongly suggests that CiaH acts as a phosphatase when acetate concentrations are high in the growth medium. Modifying acetate concentrations in C+Y medium altered CiaR-mediated expression similarly, but again less pronounced (Figure S4), indicating that CiaR is dephosphorylated under these conditions. These results are in accordance with the previous notion that CiaH acts as a phosphatase in standard C+Y medium (Halfmann et al., 2011).

The effect of acetate on CiaH activity could be caused directly by binding to CiaH, or indirectly, which would require metabolism of acetate. In the latter case, regulation should depend on AckA, since internalized acetate must be phosphorylated for further metabolism. Therefore, the *ackA* mutant strain was tested in BHI medium and in BHI with 50 mM sodium acetate. The acetate effect was virtually gone upon *ackA* inactivation (Figure S5). Therefore, the change of CiaH activity is not caused by acetate itself. Metabolism is required to produce a signal that may then be detected by CiaH.

### Response to acetate in the growth medium by variants of CiaH

Since CiaH appeared to respond to a signal produced by metabolism of acetate, it was of interest to test variants of CiaH, which have been analyzed in some detail previously (Müller et al., 2011). The majority of these *ciaH* mutants (Table 1) have been obtained in screens aimed at isolating ß-lactam resistant mutants of *S. pneumoniae* R6 (Guenzi et al., 1994; Zähner et al., 2002), but a few were also detected in *ciaH* genes of clinical isolates (Müller et al., 2011). Common to all these *ciaH* alleles, with one exception, is their ability to enhance CiaR-mediated gene expression to higher levels compared to the wild type R6. In some cases, hyperactivation of CiaR is the consequence of the mutations in *ciaH* (Müller et al., 2011).

**Figure 4:**
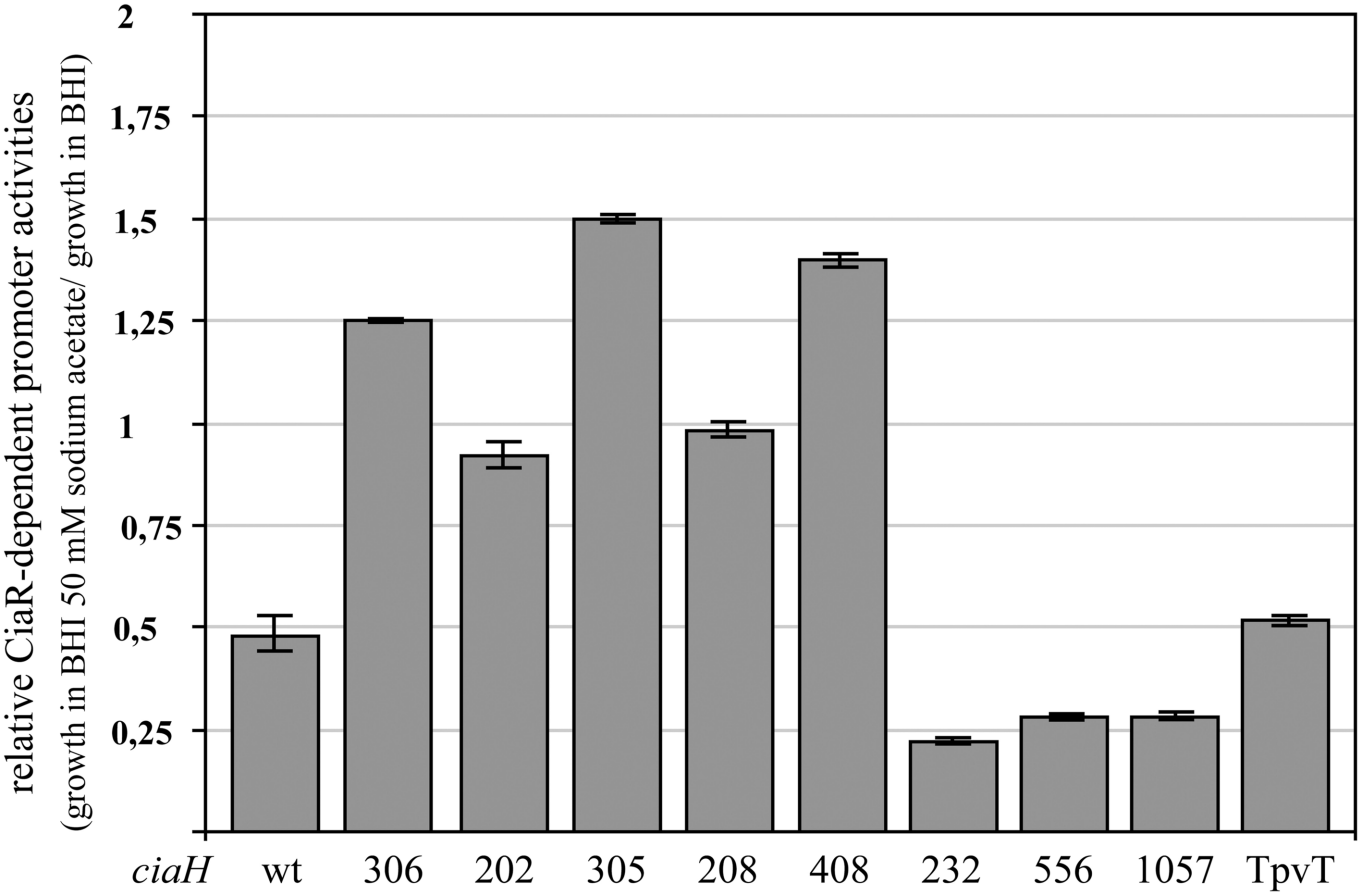
**Change of CiaR-dependent promoter activities upon acetate supplementation in strains harboring varriants of *ciaH***. The strains were grown in BHI medium containing 50 mM of sodium acetate or BHI without the addition of acetate to an OD_600_ of 0.4 and ß-galactosidase was measured. Activities of two promoters, P*_htrA_* and P_spr0931_, were determined under both growth conditions and the relative expression rate was calculated by dividing the value of the acetate supplemented culture by the value of the untreated sample. All strains contained promoter fusions in the genome (*bgaA*::*tetM*-P_spr0931_-*lacZ* or *bgaA*::*tetM*-P*_htrA_*-*lacZ*). The following strains were used (from left to right): R6, RKL168 (*ciaH306*), RKL162 (*ciaH202*), RKL163 (*ciaH305*), RKL164 (*ciaH208*), RKL165 (*ciaH408*), RKL243 (*ciaH232*), RKL245 (*ciaH556*), RKL246 (*ciaH1057*), RKL244 (*ciaH*_TpVT_). The *ciaH* allele numbers are indicated. Values of at least two independent cultures of each promoter harboring strain are shown along with standard deviations.

Promoter activities were measured in these strains in BHI and BHI with 50 mM sodium acetate. As shown in Figure 4, the response to acetate is quite variable. In the mutants obtained in the laboratory, reduction of promoter activities was not observed. In three of these strains, promoter strength was even slightly higher. Quite in contrast, three CiaH variants from clinical isolates reacted more strongly than the wild type (Figure 4), with repression rates of fourfold (*ciaH556, ciaH1057*) or even higher (*ciaH232*). The latter allele is the only one which did not enhance CiaR-mediated gene expression (Müller et al., 2011). One clinical variant (*ciaHTpVT*) behaved like the wild type. Interestingly, three CiaH variants, CiaH305, CiaH408, and CiaH232, reacting differently, have alterations in the extracytoplasmic part of the protein. Binding of an unknown factor may have been affected by these mutations. In any case, the differential response of several CiaH variants to acetate in the medium is a strong indication that the effect is specifically mediated by CiaH.

## Discussion

Genetic inactivation of genes involved in the production of AcP (Figure 1) resulted in *S. pneumoniae* strains producing drastically different amounts of AcP (Figure 2A). While the double mutant in *spxB* and *pta* produced very little or perhaps no AcP, inactivation of *ackA* alone or in combination with *pta*, raised AcP to very high levels (Figure 2A). Under latter conditions, concomitant inactivation of *ciaH* was not possible. Measuring CiaR-dependent promoters in an *spxB*-*ackA* background, a strain producing intermediate AcP amounts (Figure 2A), revealed a very strong increase in promoter activity (Figure 2C). It appears therefore, that the inability to construct *ackA*-*ciaH* and *ackA*-*pta*-*ciaH* mutants is caused by overactivation of CiaR and consequently overexpression of the CiaR regulon. And indeed, the *ackA* mutation could be combined with an inactivated *ciaR* gene (*ciaR*::*aad9*) and could be constructed in *ciaRH* wild type. In this strain, CiaH is able to reduce CiaR-dependent gene expression back to wild type levels (Figure 2B). Interestingly, in a study to determine the role of AcP in the regulation of the orphan response regulator DegU in *Listeria monocytogenes, ackA* inactivation could not be achieved unless *pta* was also inactivated (Gueriri et al., 2008).

The strains in which *ackA* could not be inactivated together with *ciaH* produce high amounts of H_2_O_2_ because of the activity of SpxB (Spellerberg et al., 1996; Pericone et al., 2003; Ramos-Montanez et al., 2008). H_2_O_2_ production alone cannot account for the lethality, since CiaRH does not affect *spxB* expression (own unpublished observations). The detrimental concerted action of H_2_O_2_ and one or more overexpressed members of the CiaR regulon remains a possibility. The stress protease HtrA appears to be one of these factors.

In contrast to the problems with *ackA* inactivation detailed above and also contrary to the strong activation of CiaR-dependent promoters in the *spxB*-*ackA* mutant strain, *ackA* mutation in a *spxB*-*pta* mutant background was readily achieved and promoted only low levels of CiaR-mediated gene expression (Figure 2C). Reducing AcP levels by sequential inactivation of *spxB, pta*, and *ackA* concomitantly lowered CiaR-dependent promoter activities tenfold in the absence of CiaH (Figure 2C). Therefore, CiaR clearly responds to AcP levels. Since phosphorylation is needed for CiaR activity and CiaR binding to promoter fragments *in vitro* is stimulated by AcP (Halfmann et al., 2011), combined evidence suggests that AcP may indeed serve as alternative source of phosphorylation for CiaR. One can always argue that an unknown histidine kinase phosphorylates CiaR by cross-talk in response to varying levels of AcP, but we consider this possibility less likely than AcP-dependent phosphorylation.

Although reduced AcP levels resulted in lower activity of CiaR, the response is not always proportional. The *ciaH*-*spxB*-*ackA* mutant produced about half of the AcP found in the wild type (Figure 2A), but promoter activities raised more than tenfold. In addition, *ackA* mutants with functional SpxB are not viable (see above). On the other hand, *ackA* inactivation did not increase promoter activities in strains producing spurious amounts of AcP (*spxB*-*pta*; Figure 2C). Thus, the effect of AckA on CiaR activity is dependent on AcP. It appears, that CiaR can use AcP as phosphodonor more efficiently in the absence of AckA. At the current stage of knowledge, it is impossible to offer conclusive explanations. An attractive hypothesis, however, would be a direct interaction between AckA and CiaR, which according to our data should lead to reduced phosphorylation of CiaR by AcP. Enzymes with additional roles in other processes are rather widespread also in bacteria (Wang et al., 2013). Prominent examples of these so-called moon-lighting proteins in bacteria are glycolytic enzymes serving also as receptors for various molecules on the bacterial surface (Wang et al., 2013). Furthermore, a number of enzymes play regulatory roles in complex physiological processes and were proposed to be called trigger enzymes (Commichau and Stülke, 2008). According to our hypothesis, AckA could be such a trigger enzyme. Interestingly, a number of bacteria possess two *ackA* genes, but the analysis of their physiological roles has just started (Chan et al., 2014; Puri et al., 2014). Intriguingly, gene duplication events are quite often associated with trigger enzymes leading to speciation of variants acting then as enzymes or as regulators (Commichau and Stülke, 2008).

The experiments to enhance CiaR-mediated gene regulation by the addition of acetate to the BHI medium constitutes another example, in which AcP levels do not correlate with promoter activities. Although the CiaR-dependent promoters were stimulated about 30-fold by the addition of 50 mM sodium acetate (Figure 3), AcP levels were not significantly elevated under these conditions. Promoter activation was completely lost (Figure S5), when a *ciaR* mutant (*ciaR152*) was tested, which expressed a non-phosphorylatable D51A variant of CiaR (Halfmann et al., 2011). Therefore, phosphorylation of CiaR is required to see the acetate effect. Without higher amounts of acetate in BHI medium, CiaR is apparently not able to use the AcP efficiently that is present in BHI grown cells. Since almost the same effects of acetate addition were observed in C+Y medium (Figure S3), this regulatory event appears to be general and not specific for BHI, where it is detected best.

To take up again our hypothesis of CiaR-AckA interaction, these results may indicate that this interaction could be regulated according to the activity of AckA or to the availability of the substrates AcP and acetate. It is clearly too early to speculate further on this type of regulation, especially because extremely little is known in *S. pneumoniae* about *ackA* expression or regulation of AckA activity, which has been shown in other organisms to be subject to allosteric control (Puri et al., 2014). In *Borrelia burgdorferi*, an orphan response regulator Rrp2 was also strongly stimulated by acetate in the growth medium, but AcP levels have not been determined (Xu et al., 2010).

A further effect of acetate addition to BHI medium was detected in the wild type (Figure 3). In the presence of CiaH, the promoter stimulating effect observed in the absence of CiaH is converted to a reduction (Figure 3). It appears that the phosphatase activity of CiaH is stimulated under these conditions. Without added acetate CiaH acts a kinase, but with 25 or 50 mM acetate as a phosphatase. Since this regulation is also dependent on AckA (Figure S5), it is very unlikely that acetate itself serves as the signal for CiaH. Rather, acetate should be metabolized to produce a signal that is sensed by CiaH. Since CiaH is lacking a recognizable intracellular sensing domain, this unknown stimulus should be located outside the cell. And indeed, three of the CiaH mutants (CiaH305, 408, 232) showing a different response compared to the wild type have amino acid substitutions in the extracytoplasmic sensing domain.

What could be the signal CiaH is responding to? Considering that CiaRH is implicated in ß-lactam resistance and maintenance of cell integrity (Guenzi et al., 1994; Mascher et al., 2006a) and that CiaR responds to AcP levels, a tempting candidate would be acetylated peptidoglycan (Vollmer, 2008). In *S*. *pneumoniae*, acetylation of peptidoglycan could be quite variable since mainly N-acetylglucosamine and to a lesser extent N-acetylmuramic acid can be deacetylated (Vollmer and Tomasz, 2000) and N-acetylmuramic acid is additionally O-acetylated (Crisostomo et al., 2006). Both processes are not essential for peptidoglycan synthesis and could be regulated and variable.

In a simple scenario, high levels of AcP, which can phosphorylate CiaR and raise its activity to high levels, would lead to more peptidoglycan acetylation, which could turn on CiaH phosphatase activity reducing CiaR activation virtually back to wild type levels. How higher peptidoglycan acetylation could be achieved by AcP is an open question. However, recent evidence clearly demonstrates that the role of AcP in acetylation at least of proteins is much more prominent than anticipated (Verdin and Ott, 2013; Weinert et al., 2013; Kuhn et al., 2014). It remains a possibility that AcP may also be a determinant in the acetylation of other compounds.

Acetylation can also be involved in the additional control of TCSs (Barak et al., 2006; Lima et al., 2011, 2012; Hu et al., 2013; Kuhn et al., 2014) and as mentioned above, AcP can serve as an acetyl donor in protein acetylation. Therefore, protein acetylation should also be altered in the *S. pneumoniae* AcP synthesis mutants described in this study. Could then acetylation explain the CiaR-related fluctuations in gene expression phenotypes rather than phosphorylation? The response of CiaR is clearly dependent on phosphorylation and CiaH is in complete control, but the strength of the change in activity may be additionally modified by protein acetylation. The genetic arguments for a special role of AckA in the process of CiaR activation remain the same. In the absence of AckA, CiaR is disproportionally active. Assuming this overactivation is caused by phosphorylation and acetylation, the underlying mechanisms would be more complicated than our proposed model.

The *S. pneumoniae* strain used throughout this study is the laboratory strain R6 derived from *S. pneumoniae* D39, which was used to identify DNA as the genetic material (Avery et al., 1944). Considering the great variety between strains of bacterial species, it is always the question whether findings in one strain also apply to others. In microarray studies of AcP production mutants in D39 it was reported that expression of several members of the CiaR regulon is reduced in a triple *spxB*-*pta*-*ackA* harboring *ciaRH* (Ramos-Montanez et al., 2010). In our study, expression went down only in the absence of *ciaH*. Strain D39 and R6 show about 80 differences, among them *spxB* (Lanie et al., 2007). But even considering the higher activity of SpxB in R6 (Belanger et al., 2004; Ramos-Montanez et al., 2008), we cannot offer an explanation for the observed difference. Curiously, two D39 variants that were kept separately for some time differed in their CiaRH-mediated gene regulation (Lanie et al., 2007). Therefore, it appears that strain-specific variations in regulatory circuits may be encountered quite frequently.

CiaRH and at least one major player in the regulatory processes analyzed in this study, SpxB, can be regarded as virulence factors (Spellerberg et al., 1996; Ibrahim et al., 2004). The human pathogen *S. pneumoniae* faces varying concentrations of oxygen and different carbohydrates in its natural host, which will inevitably change AcP levels. Since CiaRH appears to have evolved to maintain high levels of regulon expression under a variety of conditions, it would be reasonable to safeguard high expression by AcP-mediated CiaR phosphorylation, in case no appropriate signal to stimulate CiaH kinase activity is available. Since all *S. pneumoniae* genomes sequenced so far harbor *ciaRH* and *spxB*, this scenario may indeed be important for survival of *S. pneumoniae* in its natural host.

## Conflict of interest statement

The authors declare that the research was conducted in the absence of any commercial or financial relationships that could be construed as a potential conflict of interest.
