# Supplementary material for: Activity of the response regulator CiaR in mutants of Streptococcus pneumoniae R6 altered in acetyl phosphate production
Source: Front Microbiol. 2015 Jan 15;5:772. doi: 10.3389/fmicb.2014.00772 (PMC4295557; doi:10.3389/fmicb.2014.00772)
Supplement: Supplementary file 1 [file DataSheet1.PDF]

Table S1. Growth of *S. pneumoniae* acetyl phosphate production mutants.

Strain[a]	genotype	doubling time (min.)
R6	wild type	37 ± 3
RKL95	*spxB*::*ermB*	39 ± 2
RKL399	*spxB*::*ermB, pta*::*cat*	48 ± 3
RKL369	*spxB*::*ermB, pta*::*cat, ackA*::*aphIII*	52 ± 3
RKL394	*spxB*::*ermB, ackA*::*aphIII*	55 ± 2
RKL380	*pta*::*cat*	45 ± 1
RKL416	*pta*::*cat, ackA*::*aphIII*	45 ± 4
RKL379	*ackA*::*aphIII*	38 ± 3

[a] Growth rates were measured in C+Y medium with strains harboring *ciaRH* wild type.

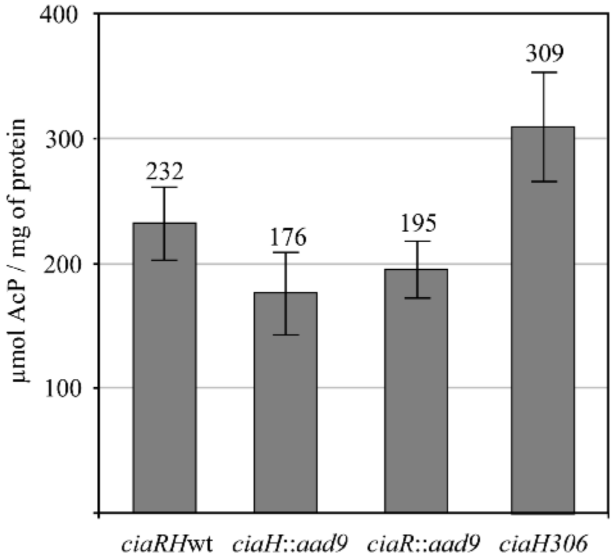

Figure S1. Acetyl phosphate production in *cia* mutant strains. The strains were grown in C+Y medium to mid-expontential growth phase (OD600 0.4) and acetyl phosphate was determined from 10 ml of the cultures. Values of at least two determinations are shown along with standard deviations. The *ciaH306* strain has a hyperactive CiaR. The differences were not significant ($p > 0.05$, two tailed Student’s t test).

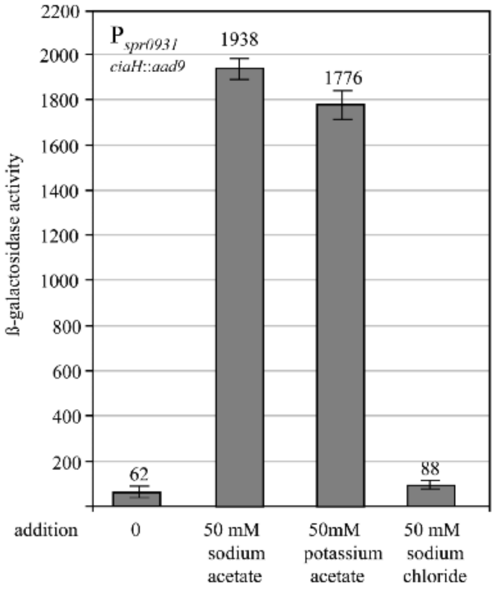

Figure S2. Effect of sodium acetate, potassium acetate, and sodium chloride additons to BHI medium on CiaR-dependent promoter activity in the absence of CiaH. The strain was grown in BHI medium containing the indicated concentrations of additions to an OD_{600} of 0.4 and ß-galactosidase was measured. Values of at least two determinations are shown along with standard deviations. The strain had a $P_{spr0931}$ promoter *lacZ* fusion in the genome (*bgaA*::*tetM*-$P_{spr0931}$-*lacZ*) and is deficient in CiaH (*ciaH*::*aad9*). ß-galactosidase units are expressed in nmol nitrophenol released per min and mg of protein.

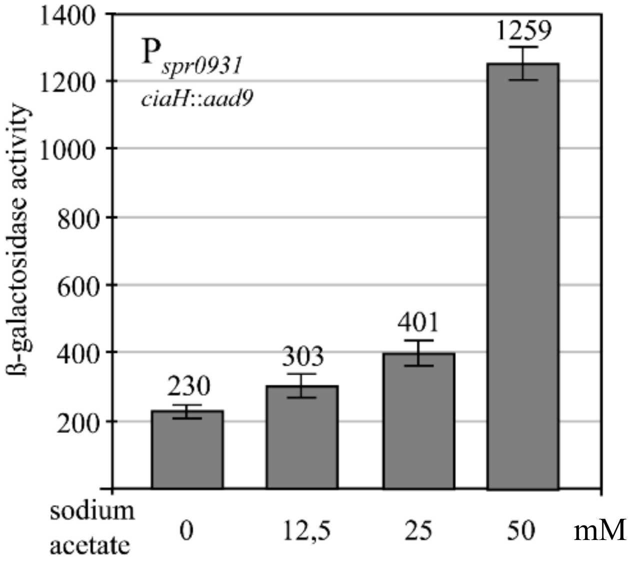

Figure S3. Effect of acetate supplementation of C+Y medium on CiaR-dependent promoter activity. The strain was grown in C+Y medium containing the indicated concentrations of sodium acetate to an OD_{600} of 0.4 and ß-galactosidase was measured. Values of at least two determinations are shown along with standard deviations. The strain had a $P_{spr0931}$ promoter *lacZ* fusion in the genome (*bgaA*::*tetM*-$P_{spr0931}$-*lacZ*) and is deficient in CiaH (*ciaH*::*aad9*). ß-galactosidase units are expressed in nmol nitrophenol released per min and mg of protein. The concentrations of sodium acetate are indicated.

.

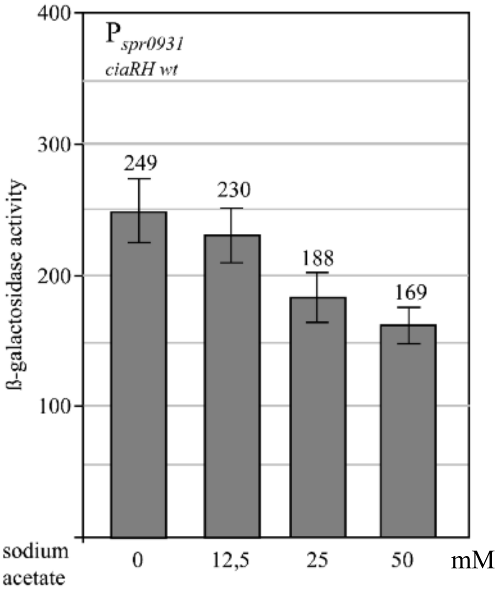

Figure S4. Effect of acetate supplementation of C+Y medium on CiaR-dependent promoter activity. The strain was grown in C+Y medium containing the indicated concentrations of sodium acetate to an OD_{600} of 0.4 and ß-galactosidase was measured. Values of at least two determinations are shown along with standard deviations. The strain had a $P_{spr0931}$ promoter *lacZ* fusion in the genome (*bgaA*::*tetM*-$P_{spr0931}$-*lacZ*) and is wild type for *ciaRH*. ß-galactosidase units are expressed in nmol nitrophenol released per min and mg of protein. The concentrations of sodium acetate are indicated.

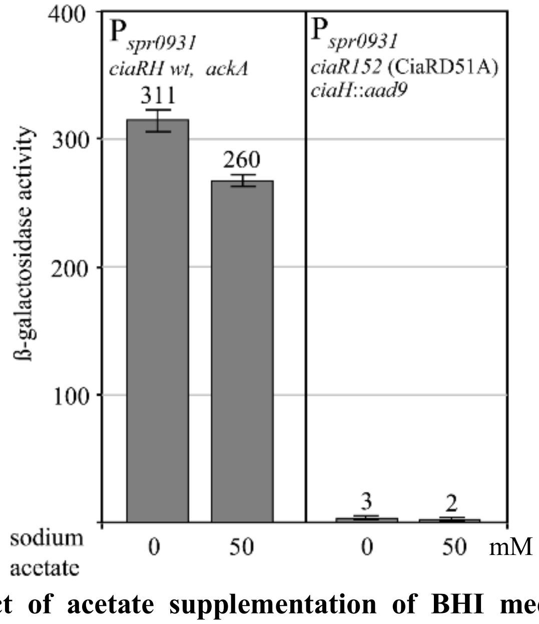

Figure S5. Effect of acetate supplementation of BHI medium on CiaR-dependent promoter activity. The strains were grown in BHI medium containing the indicated concentrations of sodium acetate to an OD_{600} of 0.4 and ß-galactosidase was measured. Values of at least two determinations are shown along with standard deviations. The strains had a $P_{spr0931}$ promoter *lacZ* fusion in the genome (*bgaA*::*tetM*-$P_{spr0931}$-*lacZ*). Values on the left represent the *ackA*::*aphIII*, *ciaRH(*wt) strain. On the right, data from the *ciaR152 (*CiaRD51A*), ciaH*::*aad9* strain are shown. ß-galactosidase units are expressed in nmol nitrophenol released per min and mg of protein. The concentrations of sodium acetate are indicated.

.
